# Experimental and Hybrid FEM/Peridynamic Study on the Fracture of Ultra-High-Performance Concretes Reinforced by Different Volume Fractions of Polyvinyl Alcohol Fibers

**DOI:** 10.3390/polym15030501

**Published:** 2023-01-18

**Authors:** Kun Zhang, Tao Ni, Jin Zhang, Wen Wang, Xi Chen, Mirco Zaccariotto, Wei Yin, Shengxue Zhu, Ugo Galvanetto

**Affiliations:** 1 Department of Transportation Engineering, Huaiyin Institute of Technology, Huaian 223003, China; 2 State Key Laboratory of Geohazard Prevention and Geoenvironment Protection, Chengdu University of Technology, Chengdu 610059, China; 3 Industrial Engineering Department, University of Padova, via Venezia 1, 35131 Padova, Italy; 4 State Key Laboratory for GeoMechanics and Deep Underground Engineering, China University of Mining & Technology, Beijing 100083, China; 5 College of Civil and Transportation Engineering, Hohai University, Nanjing 210098, China

**Keywords:** ultra-high-performance concretes, polyvinyl alcohol fiber, fiber volume fraction, three-point bending test, extended peridynamics, finite element method

## Abstract

In this study, a series of three-point bending tests were carried out with notched beam structures made of polyvinyl alcohol (PVA) fiber-reinforced ultra-high-performance concrete (UHPC) to study the effect of volume fractions of PVA fibers on the fracture characteristics of the UHPC-PVAs. Furthermore, in order to meet the increasing demand for time- and cost-saving design methods related to research and design experimentation for the UHPC structures, a relevant hybrid finite element and extended bond-based peridynamic numerical modeling approach is proposed to numerically analyze the fracture behaviors of the UHPC-PVA structures in 3D. In the proposed method, the random distribution of the fibers is considered according to their corresponding volume fractions. The predicted peak values of the applied force agree well with the experimental results, which validates the effectiveness and accuracy of the present method. Both the experimental and numerical results indicate that, increasing the PVA fiber volume fraction, the strength of the produced UHPC-PVAs will increase approximately linearly.

## 1. Introduction

Ultra-high performance concrete (UHPC) has developed as one of the most promising types of concrete in the last 25 years. This vanguard product presents both ultra-high compressive strength and remarkable durability, such as compressive strength of 150–200 MPa [1,2]. The superior performance is achieved by maximizing the packing density with very fine minerals and reactive powders. Unlike the steel bars in the reinforced concrete, the complicated design of the reinforcement layout is not necessary for UHPC elements.

It is generally accepted that the mechanical properties of UHPC can be remarkably improved with various types of fibers. The fibers made of steel, glass, polymer (such as PVA, PVC, PE), carbon, etc., mixed with high strength cement mortar could make the produced composites present quite different mechanical behaviours in the loading situation [3]. The performance is much influenced by a few parameters, e.g., the volume fraction and fiber distribution. Many authors have pointed out that steel fiber orientation can be influenced by flow patterns of mixture, rheological performance of mixture, casting methods, wall effect of formworks, extrusion of mixture and external electromagnetic field. Folgar [4] revealed that the distribution of steel fibers can be affected by the plastic viscosity and gradient of flow velocity of fresh mixture. Zhou and Uchida [5] reported that casting UHPC at the center of a slab with 1.2 m diameter can result in significant difference in steel fiber orientation value between the edge and center regions of the slab. Each of these factors could be eliminated or reduced in PVA fiber-reinforced UHPC (UHPC-PVA) material. The PVA fibers tend to develop very strong chemical bonding force with cement due to the presence of the hydroxyl group in its molecular chains [6], which causes the material to have more isotropic behaviour than other types of fiber-reinforced UHPCs. Although the PVA fiber reinforcement can increase the fracture toughness of concrete, there are still workability problems [7] to solve; an alcohol-based shrinkage reducing agent (ASRA) was first made by the authors, as reported in [8,9], with the ice-replaced mixing procedure in the production of UHPC and UHPC-PVA materials to reduce the shrinkage behavior and improve the workability.

As a fiber-reinforced composite material, it is essential to test the mechanical performance of the UHPC-PVA materials and the fracture behavior of the UHPC-PVA structures before applying a new type of UHPC-PVA to practical engineering [10,11]. Testing is the most commonly used method for revealing the mechanical properties of plain and fiber-reinforced concretes and studying their failure behaviors [12,13,14]. As reported by Yoo et al. [15], at low fiber volume fractions ($V_{f}$⩽ 1.0%), the twisted fibers provide the highest flexural strength, but they exhibit similar strength and poorer toughness than the straight fibers at a $V_{f}$ equal to or higher than 1.5%. The three-point bending test on the notched beam structures is another alternative to study the mechanical performance of UHPC-PVA structures under flexure loading [9,11]. Critical stress intensity factor, tensile strength and fracture energy can be estimated from the test results [16,17]. Although the test method is visual and useful, it has its limitation in consuming a lot of material resources and time.

In addition to experiments, numerical simulation is another effective and cost-saving method to analyze the fracture mechanisms of the fiber-reinforced structures and to evaluate their mechanical properties. In recent decades, numerical studies have been carried on the fracture characteristics of plain and fiber-reinforced concrete. Most researchers used the general finite element (FE) software with some modifications to analyze the beams and slabs made of fiber-reinforced concretes. The earliest numerical study can be found in [18], where the authors reported the simulation techniques and input parameters required to accurately simulate the strengthened concrete structures. In [19], researchers also developed a meso-scale FE model to predict the de-bonding process in fiber-reinforced concrete using a fixed angle crack model. Chen et al. [20] investigated the effects of various modeling assumptions on the interfaces between concrete, steel fiber reinforcement and shear stirrups. These authors also stressed the importance of modeling the fibers’ random distribution in the composite concrete to achieve good correlation with the measured experimental results. However, these models were only applicable in the simulations of two dimensional problems. In [21,22], ABAQUS, a general commercial FEA software, is used to perform 3D simulation of the failure of the fiber-reinforced UHPC structures under compression, flexural and tension loading by using the built-in concrete plasticity damage (CDP) model. In general, the existing relevant numerical studies did not present any techniques or recommendations to describe the crack growth process in fiber-reinforced concretes [23,24,25].

Peridynamics (PD), first proposed by Silling in 2000 [26], is a newborn non-local numerical theory, where integro-differential equations are used to describe the mechanical behavior of continuous media and discontinuities can be considered without singularities. As the earliest version of peridynamics, the bond-based peridynamics (BB-PD) theory defines the interaction by pairwise forces acting along the deformed bond, which has a limitation on the Poisson’s ration of 1/3 for plane stress and 1/4 for plane strain and 3D problems. Then, state-based peridynamic models were introduced, including ordinary and non-ordinary versions (OSB-PD and NOSB-PD), to simulate the materials with any Poisson’s ratio [27,28,29]. In recent years, PD-based computational methods have been widely used to investigate the toughening mechanisms of innovative materials [30,31,32,33]. Some relevant applications of PD-based tools to study the fracture mechanism of fiber-reinforced concretes can be found in [9,34,35,36,37,38,39,40,41]. However, in the existing literature, the fracture analysis on the fiber-reinforced concrete structures is only considered in plane stress or plane strain conditions. In addition, due to its natural non-locality, the PD-based models share the shortcoming of higher computing costs than those based on the local theory. To improve the computational efficiency and make use of the flexibilities of the PD approach in the simulation of fracture problems, coupling to the local models, such as the FE model [42,43,44,45,46,47], has become a popular and convenient choice.

The mechanical properties and fracture characteristics of the UHPCs and UHPC-PVAs produced following the manufacturing procedure reported in [8,9] have been investigated with the experimental and numerical tools. However, the cases with different PVA fiber volume fractions were not considered in the authors’ previous works. In this paper, referring to [9], the three-point bending test is used to evaluate the fracture properties of the UHPC-PVA materials. Different PVA fiber volume fractions are considered to investigate the influence on the fracture process of the UHPC-PVA structures. To comprehensively analyze the fracture behaviors of the UHPC-PVAs, a 3D hybrid FE/PD modeling approach was developed. Different from that in [9], an extended bond-based peridynamic (XBB-PD) model [29,48] equipped with an energy-based failure criterion was adopted to overcome the limitation on the Poisson ratio of the classical BB-PD model.

The main contributions of this article with regard to numerical modeling are as follows:

- The XBB-PD model is adopted to describe the deformation and fracture behaviors of UHPC-PVA structures without the limitation on the Poisson ratio;
- The PD model is coupled to the FE model to decrease the overall computational costs and maintains its flexibility in simulating crack problems;
- The discrete-level modeling procedure of the UHPC-PVA materials and structures is illustrated in detail;
- Three-dimensional simulations are carried out and the numerical results are compared to the experimental results.

In addition to that, the experimental and numerical results will explain how the strength of the UHPC-PVAs changes in cases with different volume fractions of PVA fibers. The study is a supplement to those of [8,9]. The numerical modeling approach introduced in this paper is more advanced and capable of simulating 3D crack initialization and propagation with better computational efficiency.

## 2. Experimental Program

### 2.1. Preparation of the UHPC-PVA Materials

This study focuses on the effects of the volume fractions of the PVA fiber on the fracture properties of the UHPC-PVA structures and materials. The UHPCs were prepared following the same recipe as in [9]. As listed in Table 1, the main ingredients are as follows: ASTM Type-II Portland cement, sand (approximately 1000–1500 μm in diameter), fine quartz sand (approximately 150–500 μm in diameter), EBS-S silica fume (approximately 0.1–0.5 μm in diameter), Sika polycarboxylate superplasticizer (water reducing ratio ≥ 30%), sodium laurylsulfate and polyoxyethylene nonylphenolether compounded with alcohol-based shrinkage reducing agent (ASRA, weight ratio of 2%). Alcohol was used as a solvent to combine two additives (sodium laurylsulfate and polyoxyethylene nonylphenolether), which can reduce the existence of macro-pores in the hardening matrix [8]. More information on the ingredients can be found in [8,9]. Four cases with different volume fractions of the PVA fiber ($V_{f}$), 0.5%, 1%, 1.5% and 2%, were considered to produce the UHPC-PVA materials.

A strict procedure described in [9] was carried out in the production of the UHPC-PVA materials:

- Step 1: Mix the cement, quartz sand, manufactured sand and silica fume with a prescribed mixing ratio;
- Step 2: Add ice cube and 10% water with superplasticizer and ASRA and mix for 3 min;
- Step 3: Add the remaining 90% water (mixed with PVA fibers) and process the mixture unceasingly until smooth;
- Step 4: Pour the mixture into a selected mould and vibrate for 3 min on a vibrating table;
- Step 5: Cure the specimens at room temperature for 48 h before demoulding and then cure them in a fast curing box in hot water at 90 °C for an additional 72 h.

In the mixing operation, the PVA fibers were mixed with water and then gradually added. Due to the excellent hydrophilicity, the PVA fibers can be uniformly dispersed into the hardened matrix.

### 2.2. Test Procedure

**Figure 1:**
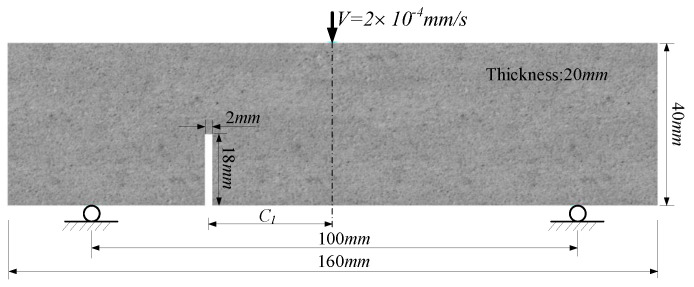
Geometry of the notched beam specimen and the loading conditions of the test.

**Figure 2:**
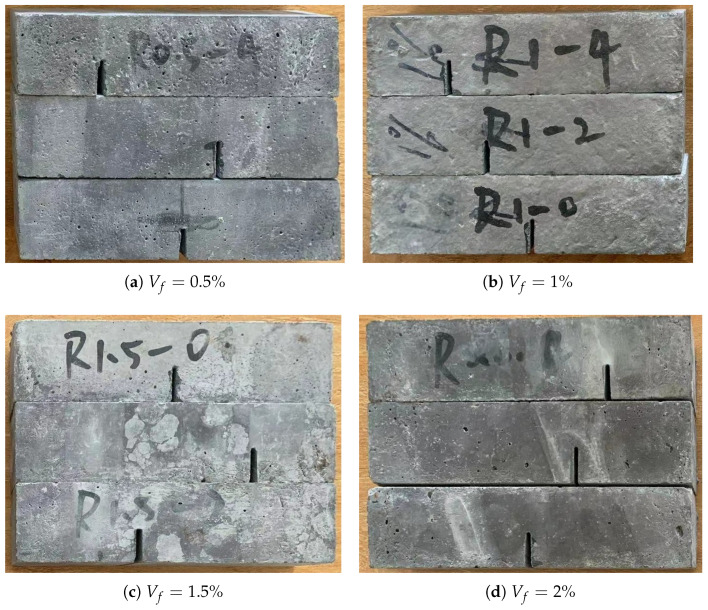
Photos of the notched UHPC-PVA beam specimens used for the three-point bending test.

In this study, a series of three-point bending tests are carried out with a notched beam specimen to evaluate the fracture properties of the produced UHPC-PVA materials. The geometry of the beam specimen and loading conditions of the test is presented in Figure 1. The cuboid specimens were produced through the designed moulds with a size of 160 mm × 40 mm × 40 mm (length × width × thickness) and then cut into the designed beam specimens with a size of 160 mm × 40mm × 20mm (length × width × thickness). The notches, with geometric parameters of $C_{l}=0\;\mathrm{mm}$, 20mm and 40mm, were fabricated by numerically controlled machine tools. All the produced notched beam specimens are shown in Figure 2a–d. As found in [8,9], the produced UHPCs and UHPC-PVAs have outstanding stable performance. Therefore, for the sake of saving material, we will use only one specimen in each case and a total of twelve specimens shown in Figure 2 will be involved in the experimental study.

**Figure 3:**
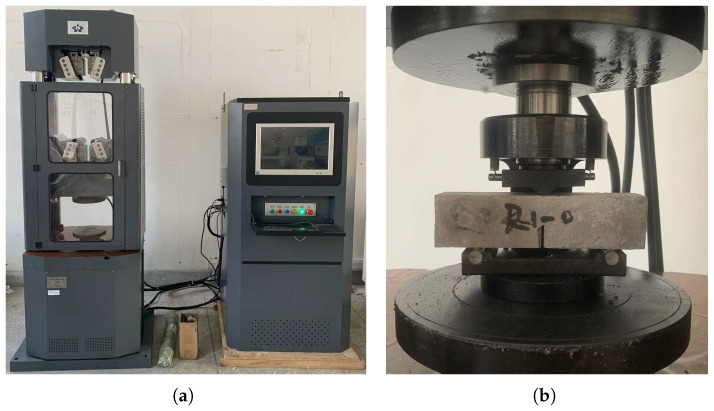
The mechanical testing system and the loading head for the three-point bending test. (**a**) Mechanical testing system, (**b**) Loading head for three-point bending test.

The tests were carried out on a electromechanical compression testing machine (WAW1000) shown in Figure 3a. The loading conditions are described as in Figure 1 and Figure 3a. The loading head forces the upper center of the beam specimens to gradually move downward at a rate of $\Delta v=2\times 10^{-4}\;\mathrm{mm}/s$ until the crack propagates and penetrates the specimens.

## 3. Numerical Model

In this section, a 3D hybrid finite element method (FEM) and extended bond-based peridynamic (XBB-PD) [29,48] modeling approach is introduced and applied to the numerical fracture analysis of UHPC-PVA materials and structures. Firstly, the governing equations of the local continuum model and the XBB-PD model are summarized. Subsequently, the model discretization and numerical implementation, including the discrete-level modeling procedure for the UHPC-PVAs, are described in detail.

### 3.1. Summary of the Mechanical Models

#### 3.1.1. Governing Equations of the Local Continuum Model

In the classical continuum mechanics, the equation of motion can be expressed as:

(1) $$\rho \ddot{u}=\nabla \cdot \sigma +b$$

where $\ddot{u}$ is the acceleration and σ is the stress tensor, b is the external force density. Under the assumption of small deformation, the stress tensor can be obtained as:

(2) σ=C:ε

where C is the elasticity tensor, ε is the strain tensor. Considering the definition of strains, if the components of the continuous displacement field in the *x*, *y* and *z* directions are defined as *u*, *v* and *w*, respectively, the strain components can be given as:

(3) $$\left\{\begin{array}{ccc} \varepsilon_{11}=\frac{\partial u}{\partial x}; & \varepsilon_{22}=\frac{\partial v}{\partial y}; & \varepsilon_{33}=\frac{\partial w}{\partial z}\\ \varepsilon_{21}=\varepsilon_{12}=\frac{\partial v}{\partial x}+\frac{\partial u}{\partial y}; & \varepsilon_{32}=\varepsilon_{23}=\frac{\partial w}{\partial y}+\frac{\partial v}{\partial z}; & \varepsilon_{31}=\varepsilon_{13}=\frac{\partial w}{\partial x}+\frac{\partial u}{\partial z} \end{array}\right.$$

#### 3.1.2. Extended Bond-Based Peridynamic Model

**Figure 4:**
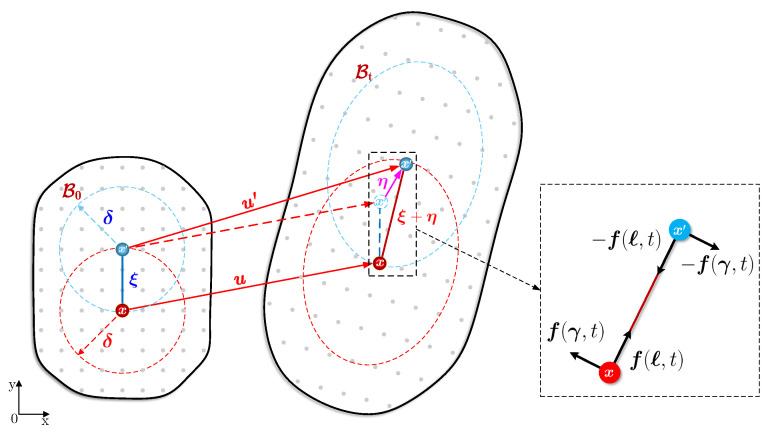
Schematic diagram of the extended bond-based peridynamic model.

As shown in Figure 4, a body B, marked as $B_{0}$ and $B_{t}$ in the initial and deformed configurations, governed by the PD model, is usually seen to be composed of a series of material points. We can assume that x is a point in B interacting with all the other points over a prescribed domain $H_{x}$. If point $x^{\prime }$ is a point within the domain $H_{x}$, the relative position of $x^{\prime }$ to x in the initial configuration can be described as:

(4) $$\xi =x^{\prime }-x$$

Then, $H_{x}$, the so-called neighbourhood, is usually a sphere space in 3D and a circle surface in 2D, which can be described as a radius of length δ (the horizon radius) and mathematically defined as:

(5) $$H_{x}=H\left(x,\delta \right)=\left\{∥\xi ∥\leq \delta :x^{\prime }\in B\right\}$$

where $∥\cdot ∥$ denotes the Euclidean norm.

In the deformed configuration, the points x and $x^{\prime }$ will be displaced by u and $u^{\prime }$, respectively. Consequently, the relative displacement vector between the two points can be given as:

(6) $$\eta =u^{\prime }-u$$

and therefore the relative position vector in the deformed configuration can be given as ξ+η.

In the extended bond-based PD theory, the equation of motion at point x can be expressed as:

(7) $$\rho \ddot{u}\left(x,t\right)=\int_{H_{x}}f\left(\eta ,\xi ,t\right)dV_{x^{\prime }}+b\left(x,t\right)$$

where ρ is the mass density, $\ddot{u}\left(x,t\right)$ is the acceleration of point x at time instant *t*, $dV_{x^{\prime }}$ is the mass volume associated with point $x^{\prime }$, b(x,t) is the body force density to point x applied by the external loads. f(η,ξ,t) is the pairwise force density exerted to point x by the deformed bond, containing two contributions from the longitudinal and tangential deformations (see Figure 4), which can be expressed by [48]:

(8) f(η,ξ,t)=cℓ(η,ξ,t)n+κγ(η,ξ,t)

where *c* and κ are the normal and tangential micro moduli of the bond, ℓ(η,ξ,t) and γ(η,ξ,t) are the longitudinal and tangential deformations of the bond. n is the unit directional vector along the deformed bond and its formulae can be given as:

(9) $$n=\frac{\eta +\xi }{∥\eta +\xi ∥}$$

The expressions of the normal and tangential micro moduli can be obtained from a comparison with the strain energy of local continuum mechanics for homogeneous deformation [29]. Their expressions in terms of the elastic constants of Young’s modulus *E* and Poisson’s ratio ν of the material can be obtained by:

(10) $$\left\{\begin{array}{ccc} c & = & \frac{6E}{\pi \delta^{4}\left(1-2\nu \right)}\\ \kappa & = & \frac{6E\left(1-4v\right)}{\pi \delta^{4}\left(1+\nu \right)\left(1-2\nu \right)} \end{array}\right.$$

Referring to [29,48], based on the Cauchy–Born criterion, the relationships between the local deformations of the bond and the macroscopic strain can be constructed as:

(11) ℓ=n·ε·n

and

(12) γ=n·ε·(I−n⊗n)

where ε is the strain tensor and I is the second order unit tensor.

Furthermore, the longitudinal deformation can also be formulated based on the geometrical analyses [29]:

(13) $$\ell =\frac{1}{\xi }\eta \cdot n$$

which is more efficient than Equation (11) and in this paper this formulae will be used to evaluate the longitudinal deformation.

To describe the material failure and crack propagation, a bond failure criterion is essential for the PD models. The critical bond-stretch criterion is the first introduced and most commonly used criterion to judge the bond breakage in the classical bond-based and state-based PD simulations. However, there are two deformation components in the micro-constitutive law and a failure criterion associated only with the bond stretch (longitudinal deformation) will not be able to reflect the effect of the tangential deformation on the failure behaviours. Thus, inspired by [49], an energy-based failure criterion will be adopted for the XBB-PD model to simulate the fracture problems.

The strain energy density stored in the deformed bond ξ can be computed by:

(14) $$w\left(\xi \right)=w\left(\ell ,\gamma \right)=\frac{1}{2}c\ell^{2}\xi +\frac{1}{2}\kappa \gamma \cdot \gamma \xi$$

Following the derivations in [48,49], the critical strain energy density of the bond can be given as:

(15) $$w_{c}=\frac{4G_{c}}{\pi \delta^{4}}$$

which means that the bond will be broken when its strain energy density w(ξ) becomes greater than $w_{c}$ and accordingly, a scalar variable is defined to indicate the connection state of the bond [50,51]:

(16) $$\%\left(\xi \right)=\left\{\begin{array}{ccc} 1 & , & \mathrm{if}\;w\left(\xi \right)<w_{c}\\ 0 & , & \mathrm{otherwise} \end{array}\right.$$

Consequently, the damage level at point x can be defined as:

(17) $$\varphi_{x}=1-\frac{\int_{H_{x}}\%\left(\xi \right)dV_{x^{\prime }}}{\int_{H_{x}}dV_{x^{\prime }}}$$

where $\varphi_{x}\in \left[0,1\right]$ and the cracks are usually identified wherever $\varphi_{x}⩾0.5$.

### 3.2. Discretization and Numerical Implementation

To obtain an acceptable numerical solution, a suitable discretization process is necessary. This section will introduce the numerical discretization of the FE and PD equations and their coupled modeling strategy for the UHPC-PVA materials and structures. In order to obtained a quasi-static solution of the coupled model and compare with the experimental observations, the adaptive dynamic relaxation algorithm is also briefly summarized.

#### 3.2.1. FEM Discretization of the Governing Equations Based on Local Theory

The Galerkin finite element method [52] is adopted here to discretize the governing equations of the continuum mechanical model. The FE equation of motion can be written as the following matrix form:

(18) $$M^{FEM}\ddot{U}+K^{FEM}U=F$$

where $M^{FEM}$ and $K^{FEM}$ are the mass and stiffness matrices of the FE domain. Given the shape function $N_{u}$ for the displacement, the stiffness matrices in Equation (18) can be obtained by:

(19) $$M^{FEM}=\int_{\Omega }N_{u}^{T}\rho N_{u}d\Omega$$

and

(20) $$K^{FEM}=\int_{\Omega }{\left({\mathrm{LN}}_{u}\right)}^{T}D\left({\mathrm{LN}}_{u}\right)d\Omega$$

in which L is the differential operator defined as:

(21) $$L=\left[\begin{array}{ccc} \frac{\partial }{\partial x} & 0 & 0\\ 0 & \frac{\partial }{\partial y} & 0\\ 0 & 0 & \frac{\partial }{\partial z}\\ \frac{\partial }{\partial y} & \frac{\partial }{\partial x} & 0\\ 0 & \frac{\partial }{\partial z} & \frac{\partial }{\partial y}\\ \frac{\partial }{\partial z} & 0 & \frac{\partial }{\partial x} \end{array}\right]$$

and D is the elastic matrix given as:

(22) $$D=\frac{E(1-v)}{\left(1+v)(1-2v\right)}\left[\begin{array}{cccccc} 1 & \frac{v}{1-v} & \frac{v}{1-v} & 0 & 0 & 0\\ \frac{v}{1-v} & 1 & \frac{v}{1-v} & 0 & 0 & 0\\ \frac{v}{1-v} & \frac{v}{1-v} & 1 & 0 & 0 & 0\\ 0 & 0 & 0 & \frac{1-2v}{2(1-v)} & 0 & 0\\ 0 & 0 & 0 & 0 & \frac{1-2v}{2(1-v)} & 0\\ 0 & 0 & 0 & 0 & 0 & \frac{1-2v}{2(1-v)} \end{array}\right]$$

where *E* and ν are the Young’s modulus and Poisson’s ratio of the material.

#### 3.2.2. Discretization of the XBB-PD Equations

After discretization, the spatial integrals in the XBB-PD equations will be written into forms of summation over nodes in the neighbourhood. Then, the equation of motion of node $x_{i}$ at time *t* will be:

(23) $$\rho {\ddot{u}}_{i}^{t}=\sum_{j=1}^{N_{H_{i}}}f^{t}\left(\xi_{ij}\right)V_{j}+b_{i}^{t}$$

where $N_{H_{i}}$ is the number of family nodes in $x_{i}$’s horizon. $x_{j}$ represents $x_{i}$’s family node and $V_{j}$ is its volume. $b_{i}^{t}$ is the body force density of node $x_{i}$. $f^{t}\left(\xi_{ij}\right)$ is the internal force density exerted to node $x_{i}$ via the deformed bond $\xi_{ij}$, which can be computed by:

(24) $$f^{t}\left(\xi_{ij}\right)=\left[f_{ij}\right]=\left[f_{ij}^{\ell }\right]+\left[f_{ij}^{\gamma }\right]=c\ell_{ij}\left[n_{ij}\right]+\kappa \left[\gamma_{ij}\right]$$

in which $\ell_{ij}$ and $\left[\gamma_{ij}\right]$ are the longitudinal and tangential deformation components of the bond $\xi_{ij}$ and $\left[n_{ij}\right]$ is the longitudinal unit vector. The two vectors can be defined as:

(25) $$\begin{array}{ccc} \left[n_{ij}\right]={\left[n_{1}\;\;n_{2}\;\;n_{3}\right]}^{T}\; & \mathrm{and} & \left[\gamma_{ij}\right]={\left[\gamma_{1}\;\;\gamma_{2}\;\;\gamma_{3}\right]}^{T} \end{array}$$

if the displacement vectors of nodes $x_{i}$ and $x_{j}$ are given as $\left[U_{i}\right]={\left[U_{i}^{1}\;\;U_{i}^{2}\;\;U_{i}^{3}\right]}^{T}$ and $\left[U_{j}\right]={\left[U_{j}^{1}\;\;U_{j}^{2}\;\;U_{j}^{3}\right]}^{T}$. According to Equation (13), the stretch (longitudinal deformation) of the bond $\xi_{ij}$ can be obtained by:

(26) $$\ell_{ij}=\left[C_{ij}^{\ell }\right]\left[\begin{array}{c} U_{i}\\ U_{j} \end{array}\right]$$

where $\left[C_{ij}^{\ell }\right]$ can be given as:

(27) $$\left[C_{ij}^{\ell }\right]=\frac{1}{\xi_{ij}}\left[\begin{array}{cccccc} -n_{1} & -n_{2} & -n_{3} & n_{1} & n_{2} & n_{3} \end{array}\right]$$

Marking the strains at nodes $x_{i}$ and $x_{j}$ as $\left[\varepsilon_{i}\right]$ and $\left[\varepsilon_{j}\right]$, they can be written in vector forms as:

(28) $$\begin{array}{cccccc} \left[\varepsilon_{i}\right]={\left[\varepsilon_{i}^{1}\;\;\varepsilon_{i}^{2}\;\;\varepsilon_{i}^{3}\;\;\varepsilon_{i}^{12}\;\;\varepsilon_{i}^{13}\;\;\varepsilon_{i}^{23}\right]}^{T} & \mathrm{and} & \left[\varepsilon_{j}\right]={\left[\varepsilon_{j}^{1}\;\;\varepsilon_{j}^{2}\;\;\varepsilon_{j}^{3}\;\;\varepsilon_{j}^{12}\;\;\varepsilon_{j}^{13}\;\;\varepsilon_{j}^{23}\right]}^{T} & , & \; & \end{array}$$

respectively.

According to Equation (12), the tangential deformation vector of the bond $\xi_{ij}$ can be obtained by:

(29) $$\left[\gamma_{ij}\right]=\left[C_{ij}^{\gamma }\right]\left[\varepsilon_{ij}\right]$$

where $\left[C_{ij}^{\gamma }\right]$ is given as:

(30) $$\left[C_{ij}^{\gamma }\right]=\left[\begin{array}{cccccc} n_{1}-n_{1}^{3} & -n_{1}n_{2}^{2} & -n_{1}n_{3}^{2} & n_{2}-2n_{1}^{2}n_{2}\; & n_{3}-2n_{1}^{2}n_{3} & -2n_{1}n_{2}n_{3}\\ -n_{1}^{2}n_{2} & n_{2}-n_{2}^{3} & -n_{2}n_{3}^{2} & n_{1}-2n_{1}n_{2}^{2}\; & -2n_{1}n_{2}n_{3} & n_{3}-2n_{2}^{2}n_{3}\\ -n_{1}^{2}n_{3} & -n_{2}^{2}n_{3} & n_{3}-n_{3}^{3} & -2n_{1}n_{2}n_{3} & n_{1}-2n_{1}n_{3}^{2} & n_{2}-2n_{2}n_{3}^{2} \end{array}\right]$$

and $\left[\varepsilon_{ij}\right]$ is the average strain of the bond $\xi_{ij}$ defined as:

(31) $$\left[\varepsilon_{ij}\right]=\frac{\left[\varepsilon_{i}\right]+\left[\varepsilon_{j}\right]}{2}$$

Based on the above notions, the PD force density exerted to node $x_{i}$ by the bond $\xi_{ij}$ can be obtained by:

(32) $$\left[f_{ij}\right]=c\left[n_{ij}\right]\left[C_{ij}^{\ell }\right]\left[\begin{array}{c} U_{i}\\ U_{j} \end{array}\right]+\frac{1}{2}\kappa \left[\begin{array}{cc} C_{ij}^{\gamma } & C_{ij}^{\gamma } \end{array}\right]\left[\begin{array}{c} \varepsilon_{i}\\ \varepsilon_{j} \end{array}\right]$$

Consequently, the equations of motion of the XBB-PD model can be assembled and written in the following matrix form:

(33) $$M^{PD}\ddot{U}+cN^{\ell }C^{\ell }U+\kappa C^{\gamma }E=F$$

where $M^{PD}$ is the diagonal mass matrix, $N^{\ell }$, $C^{\ell }$ and $C^{\gamma }$ are matrices assembled from the matrices of Equations (25), (27) and (30). $\ddot{U}$, U, E and F are the acceleration, displacement, strain and force vectors of the nodes, respectively.

As described in Equation (3), the strain components are the spatial partial derivatives of the displacement field. In the PD framework, the peridynamic differential operator (PDDO) proposed in [53] can be used to evaluate derivatives. Referring to [48], the global relationship between the displacement field and the strain field can be written in the following form:

(34) E=GU

where G is the non-local strain coefficient matrix [48].

Therefore, substitution of Equation (34) into Equation (33) converts the PD equations of motion into the following concise form:

(35) $$M^{PD}\ddot{U}+K^{PD}U=F$$

where $K^{PD}=cN^{\ell }C^{\ell }+\kappa C^{\gamma }G$ is the assembled stiffness matrix of the XBB-PD model.

#### 3.2.3. Hybrid FEM and PD Modelling Approach for the UHPC-PVA Materials and Structures

**Figure 5:**
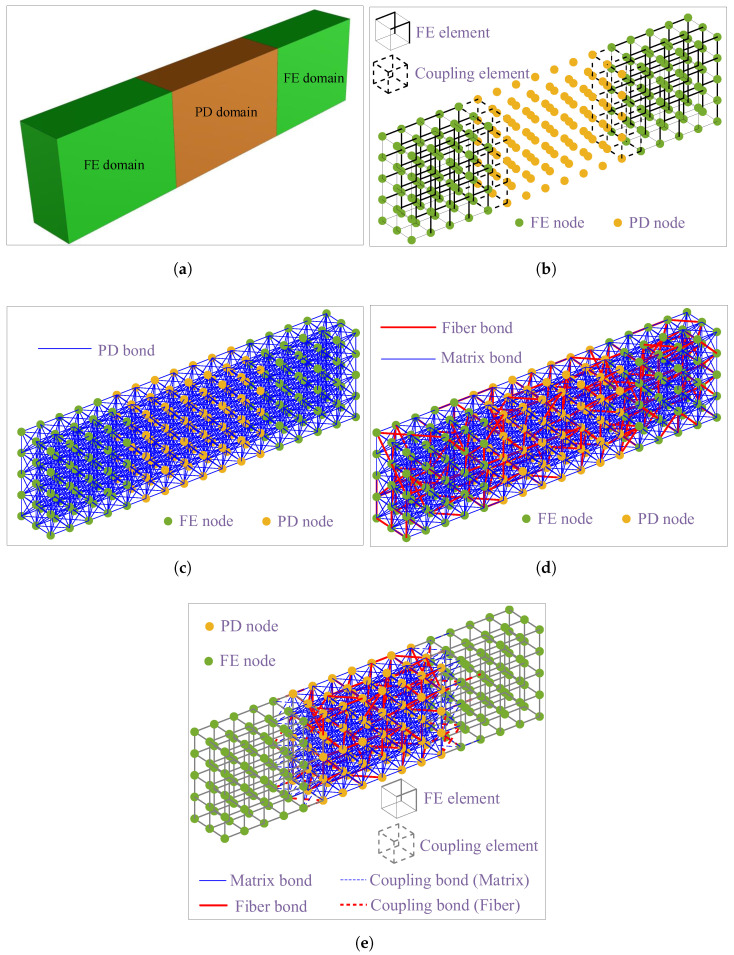
The diagrammatic sketch for the illustration of the hybrid FE/PD modeling procedure of the UHPC-PVA structures. Note that the size of the grid does not represent the real one used in the simulations. (**a**) Modelling schematic of a beam specimen; (**b**) Step 1: the hybrid FE/PD discretization; (**c**) Step 2: PD bond connections for all nodes; (**d**) Step 3: random selection of fiber bonds; (**e**) Step 4: the complete hybrid FE/PD model.

The approach introduced in [9] is adopted here to model the UHPC-PVA materials and structures, where the interaction between the PVA fibers and the matrix is considered in the discrete level. The hybrid FEM/PD modeling procedure of UHPC-PVA materials and structures is described in Figure 5.

As shown in Figure 5a, the beam specimen is divided into FE and PD domains. Then, the hybrid model will be generated according to the following steps:

- Step 1: Discretize the FE and PD domains by using the FE mesh and PD grid with the same grid size; see Figure 5b;
- Step 2: Generate the PD bonds connecting all the FE and PD nodes; see Figure 5c;
- Step 3: Randomly select a certain number of bonds and set their parameters as the mechanical parameters of the PVA material; then, the rest are the matrix bonds with the mechanical parameters of the UHPC materials. The obtained model is shown in Figure 5d. The ratio of the total length of fiber bonds to the total length of all bonds, which is called the global numerical volume fraction of PVA fibers ($V_{f}^{g}$), is approximately equal to the volume fraction of fibers in the modeled UHPC-PVA material;
- Step 4: Determine the final FE/PD model, as shown in Figure 5e, where the reinforcement at the FE and coupling elements is considered based on the local numerical volume fraction of PVA fibers ($V_{f}^{l}$).

The global numerical volume fraction of PVA fibers can be calculated by:

(36) $$V_{f}^{g}=\frac{\sum_{i=1}^{N_{n}}\sum_{j=1}^{N_{i}^{f}}∥\xi_{ij}∥}{\sum_{i=1}^{N_{n}}\sum_{j=1}^{N_{i}}∥\xi_{ij}∥}$$

where $N_{n}$ is the number of nodes in the discrete model; $N_{H_{i}}$ is the number of $x_{i}$’s family nodes, while $N_{i}^{f}$ is the number of $x_{i}$’s family nodes connected by the fiber bonds. Consequently, the local numerical volume fraction of PVA fibers at node $x_{i}$ can be obtained by:

(37) $$V_{f}^{l_{i}}=\frac{\sum_{j=1}^{N_{i}^{f}}∥\xi_{ij}∥}{\sum_{j=1}^{N_{i}}∥\xi_{ij}∥}$$

Given the $V_{f}^{l}$ value at each node, the reinforcement of the PVA fibers on the UHPC matrix will be expressed by:

(38) $$P_{i}=P_{m}\left(1-V_{f}^{l_{i}}\right)+P_{f}V_{f}^{l_{i}}$$

where $P_{i}$ represents the mechanical parameters at node *i*; $P_{m}$ and $P_{f}$ are the parameters of the UHPC and PVA materials, respectively. Therefore, the reinforcement of the PVA fibers on the matrix will be considered in the calculation of the elastic matrix of Equation (22).

The system matrix of the hybrid FEM and PD model can be expressed by:

(39) $$M^{Coup}\ddot{U}+K^{Coup}U=F$$

Note that, instead of the formation in Equation (19), the mass density matrix of the FE domain will use a diagonal form to maintain consistency with the PD domain.

#### 3.2.4. Quasi-Static Solution Algorithm

The adaptive dynamic relaxation (ADR) algorithm was first proposed by Underwood in [54] to obtain the quasi-static solutions of non-linear problems. Later in [45,55,56,57], the ADR algorithm was successfully applied to solve the static or quasi-static solutions of PD models.

In accordance with our experience in [9], the tests described above adopted a quasi-static loading process. Therefore, the ADR algorithm will be equipped with the hybrid FEM/XBB-PD model to analyze the fracture process of the UHPC-PVA beams under the three-point bending load.

By introducing a damping term, the global governing equation of the hybrid model at the $n^{th}$ time increment can be written in the following form:

(40) $$M{\ddot{U}}^{n}+C_{d}{\dot{U}}^{n}+KU^{n}=F^{n}$$

where M, $C_{d}$ and K are the fictitious mass, damping and stiffness matrices. F is the external force vector. Subsequently, the central time difference form will be adopted in the ADR algorithm and the displacement at the ${\left(n+1\right)}^{th}$ iteration can be obtained by:

(41) $$U^{n+1}=U^{n}+{\dot{U}}^{n+1/2}\Delta t$$

where the velocity at the ${\left(n+1/2\right)}^{th}$ iteration can be calculated by:

(42) $${\dot{U}}^{n+1/2}=\frac{M/\Delta t-\frac{1}{2}C_{d}}{M/\Delta t+\frac{1}{2}C_{d}}{\dot{U}}^{n-1/2}+\frac{\left[F-KU^{n}\right]\Delta t}{M/\Delta t+\frac{1}{2}C_{d}}$$

where Δt is the time increment.

In order to solve Equation (42) explicitly, a diagonal fictitious mass matrix is required. $M_{ii}$ is the $i^{th}$ principal value of the fictitious mass matrix M, which needs to satisfy the following inequality:

(43) $$M_{ii}⩾\frac{1}{4}\Delta t^{2}\sum_{j}\left|K_{ij}\right|$$

where $K_{ij}$ are the elements of the global stiffness matrix K. To simplify the time integral process, the damping matrix is usually defined as multiples of the fictitious mass matrix:

(44) $$C_{d}=c_{d}M$$

where $c_{d}$ is a system damping coefficient that needs to be updated during the iterations. The value of $c_{d}$ at the $n^{th}$ iteration can be computed by:

(45) $$c_{d}^{n}=2\sqrt{\frac{{\left(U^{n}\right)}^{T}K_{t}^{n}U^{n}}{{\left(U^{n}\right)}^{T}{\mathrm{MU}}^{n}}}$$

where $K_{t}^{n}$ is the “local” diagonal tangent stiffness matrix at the $n^{th}$ iteration and its diagonal entries are defined as:

(46) $${\left(K_{t}^{n}\right)}_{ii}=\frac{KU^{n}-KU^{n-1}}{{\dot{U}}^{n-1/2}\Delta t}$$

Substituting Equation (44) into Equation (42), Equation (42) can be rewritten as follows:

(47) $${\dot{U}}^{n+1/2}=\frac{2-c_{d}^{n}\Delta t}{2+c_{d}^{n}\Delta t}{\dot{U}}^{n-1/2}+\frac{2\left[F-KU^{n}\right]\Delta t}{\left(2+c_{d}^{n}\Delta t\right)M}$$

In addition, the iteration starts with:

(48) $${\dot{U}}^{1/2}=\frac{\left[F-KU^{0}\right]\Delta t}{2M}$$

#### 3.2.5. The Model Parameters and Settings in the Simulations

In this section, the determination of the discretization and mechanical parameters needed in the numerical simulations is described.

According to the experimental results in [8,9] and the characteristics of the XBB-PD model, the mechanical parameters of the produced UHPC material adopted in the numerical simulations are taken as Young’s modulus: $E_{m}=34.5\;\mathrm{GPa}$; Poisson’s ratio: $\nu_{m}=0.1$; fracture energy density: $G_{cm}=90\;{J/m}^{2}$ (measured by the approach introduced in [58]). On the other hand, the mechanical parameters of the PVA materials provided by the manufacturer are given as Young’s modulus: $E_{f}=100\;\mathrm{GPa}$; Poisson’s ratio: $\nu_{f}=0.22$; fracture energy density: $G_{cf}=8000\;{J/m}^{2}$.

In [9], the produced UHPC-PVA materials were seen as a type of composite material. In order to keep the smoothness of the strain field in the PD simulation of such a composite material, the horizon radius used for the PD discretization should conform to the following inequality:

(49) $$\delta ⩽\sqrt{\frac{E_{f}h_{f}h_{m}^{2}}{3\mu_{m}\left(h_{f}+h_{m}\right)}}$$

where $h_{f}$ and $h_{m}$ represent the geometrically characteristic lengths of the matrix and fiber materials; $\mu_{m}$ is the shear modulus of the matrix material. As we stated in [9], the geometrically characteristic lengths can be taken as $h_{f}=20\;\mathrm{mm}$ and $h_{m}=1.5\;\mathrm{mm}$, respectively. Given the Young’s modulus of the PVAs $E_{f}=100\;\mathrm{GPa}$ and the shear modulus of the UHPCs $\mu_{m}=E_{m}/\left(1+2\nu_{m}\right)=28.75\;\mathrm{GPa}$, using Equation (49), the horizon radius should satisfy δ⩽1.558mm and δ=1.5mm could be a convenient choice.

**Figure 6:**
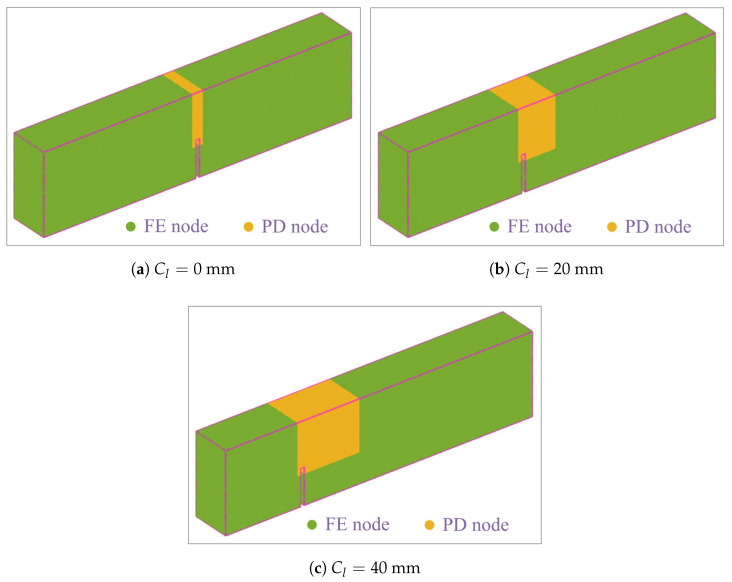
The discrete models used in the 3D simulations.

In [9], the *m*-ratio was taken as m=5 for the 2D modeling of UHPC-PVA structures. However, the 3D condition is considered in this paper. For the purpose of compromise between accuracy and computational cost, the *m* ratio is adopted here as m=3, then the grid size is obtained as Δx=δ/m=0.5mm. The discrete models for the three cases in the experiments are shown in Figure 6a–c. The number of total nodes is 1,061,613 in the hybrid models. The discretization information is presented in detail in Table 2.

The ADR algorithm is sensitive to the value of Δt used in the time integration [9,45]. In [9], a numerical test was performed with a 2D model to determine the proper value of Δt to find the similar quasi-static characteristics for the experimental observations. Referring to that, $\Delta t=5\times 10^{-3}s$ could be the most secure value and will be used in all the 3D simulations.

In order to compare with the experimental results, four different values of $V_{f}$ (= 0.5%, 1%, 1.5% and 2%) are considered. Given the loading rate of $v=2\times 10^{-4}\;\mathrm{mm}/s$ adopted in the experiments, the numbers of iterations in the simulations will be 350,000, 400,000 and 500,000 for cases 1, 2 and 3, respectively.

## 4. Experimental and Numerical Results

### 4.1. Experimental Results

**Figure 7:**
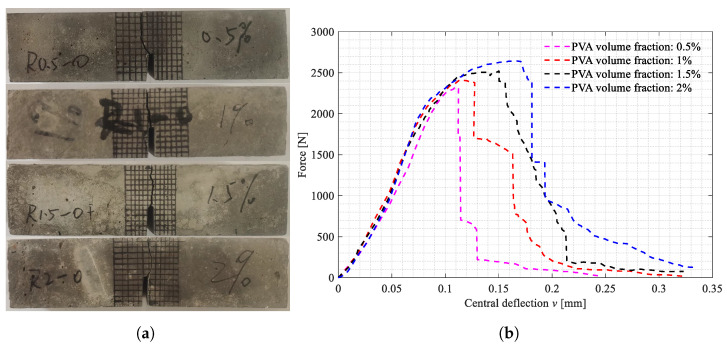
The broken beam specimens with $C_{l}=0\;\mathrm{mm}$ and the central deflection-force diagrams. (**a**) Broken specimens with $C_{l}=0\;\mathrm{mm}$; (**b**) Central deflection-force diagrams.

**Figure 8:**
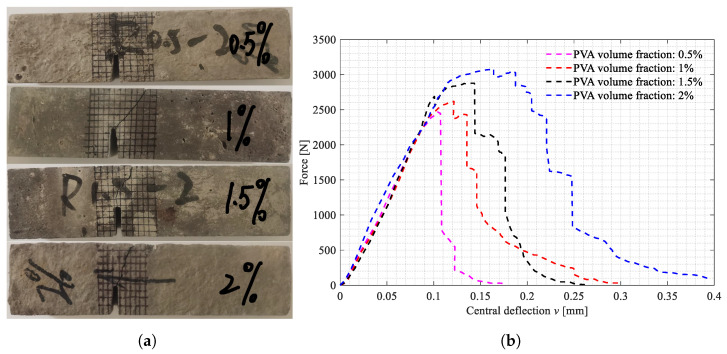
The broken beam specimens with $C_{l}=20\;\mathrm{mm}$ and the central deflection-force diagrams. (**a**) Broken specimens with $C_{l}=20\;\mathrm{mm}$; (**b**) Central deflection-force diagrams.

**Figure 9:**
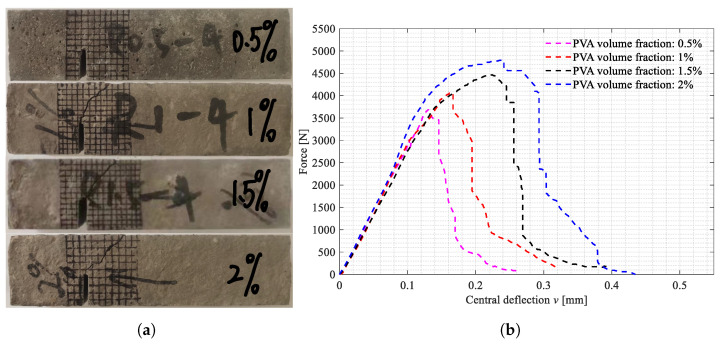
The broken beam specimens with $C_{l}=40\;\mathrm{mm}$ and the central deflection-force diagrams. (**a**) Broken specimens with $C_{l}=40\;\mathrm{mm}$; (**b**) Central deflection-force diagrams.

**Figure 10:**
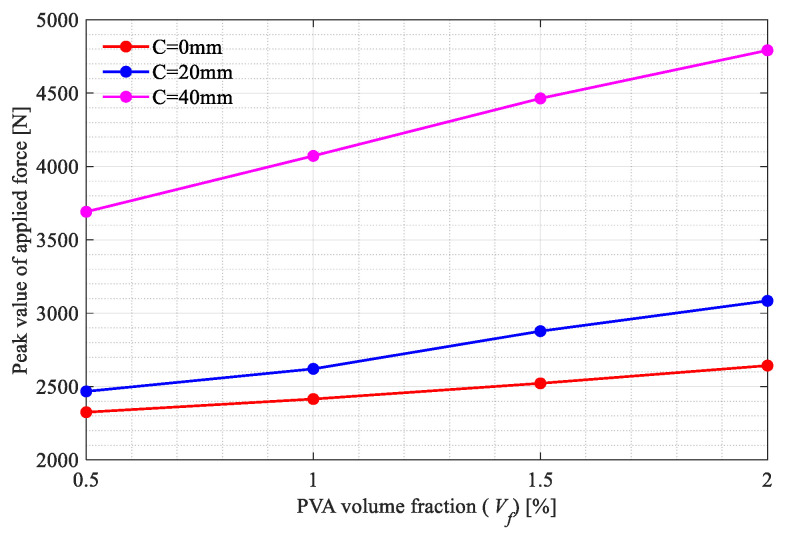
Variations in the peak force values in the tests versus the volume fractions of PVA fibers in the UHPC-PVAs.

The three-point bending tests on the beam specimens shown in Figure 2a–d were carried out. All the broken specimens are shown in Figure 7a, Figure 8a and Figure 9a. The variations of the applied loads versus the central deflections are recorded and plotted in Figure 7b, Figure 8b and Figure 9b. The shapes of the central deflection-force diagrams show different characteristics in the elastic, hardening, softening and failure stages in the cases with different PVA fiber volume fractions. In contrast, the results of the compression tests [8] and the three-point bending tests [9] performed with the produced plain UHPC materials showed a typical quasi-brittle behavior. It seems that, due to the addition of the PVA fibers and with the increase in the volume fraction, the UHPC-PVA materials gradually change from brittle to ductile. The significant toughening enhancement phenomena exist in the cases with greater PVA fiber volume fractions. Figure 10 shows the variations of the peak force values in the tests versus the volume fractions of PVA fibers mixed in the UHPC-PVAs, describing that the strength of the produced materials increases approximately linearly with PVA fiber volume fraction.

**Figure 11:**
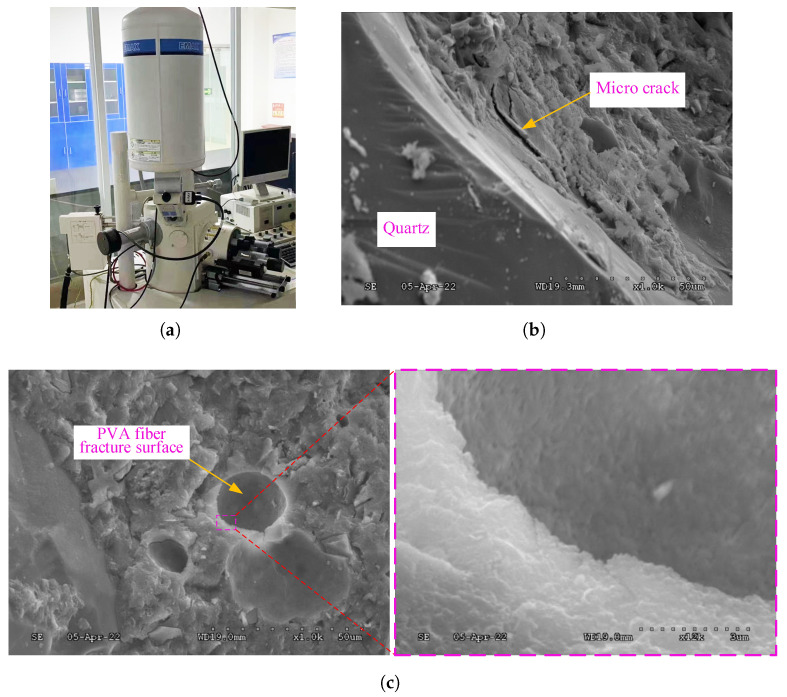
SEM micrographs of the fracture surfaces in the broken UHPC-PVA beams. (**a**) SEM; (**b**) A SEM micrograph near a quartz granule; (**c**) A SEM micrograph near a PVA fiber.

The scanning electron microscope (SEM) shown in Figure 11a is used here to study the micro characteristics of the fracture surface in the UHPC-PVA beam specimen after tests. Figure 11b,c show two SEM micrographs near a PVA fiber and a quartz granule on the fracture surface. As shown in Figure 11b, during the fracture advancement, there is a granular peeling phenomenon near the quartzite–cement interface, but the PVA–cement interface is smooth (see Figure 11c). The difference suggests that the chemical bonding between the PVA fibers and cement matrix is much stronger than that of quartzite granules. This also explains why the PVA fibers can reinforce the produced UHPC materials. On the other hand, as stressed in [9], the PVA fibers were broken at the fracture surfaces and no pulling-out phenomena were observed, which justifies the proposed modeling approach for the produced UHPC-PVA materials and structures.

### 4.2. Numerical Results

**Figure 12:**
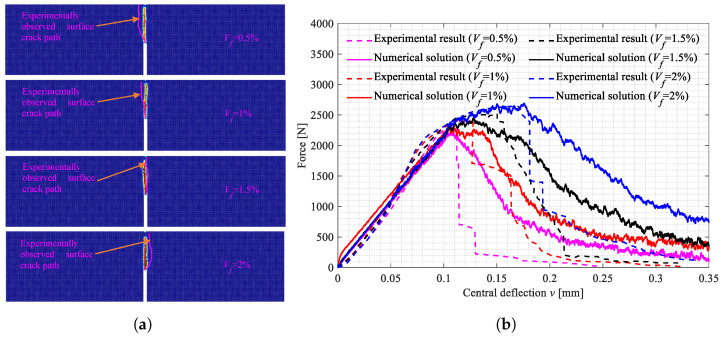
The crack patterns in the beam specimens with $C_{l}=0\;\mathrm{mm}$ and the central deflection-force diagrams. (**a**) Surface crack patterns; (**b**) Central deflection-force diagrams.

**Figure 13:**
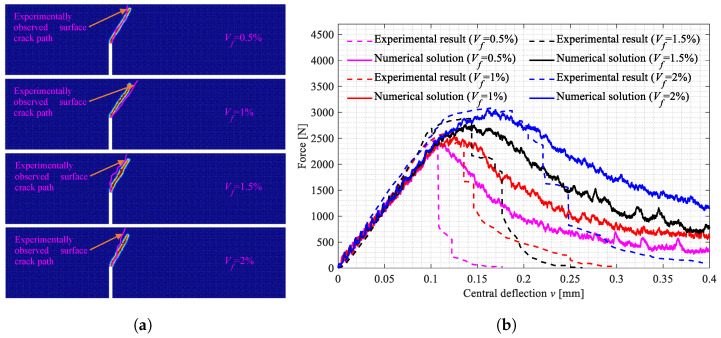
The crack patterns in the beam specimens with $C_{l}=20\;\mathrm{mm}$ and the central deflection-force diagrams. (**a**) Surface crack patterns; (**b**) Central deflection-force diagrams.

**Figure 14:**
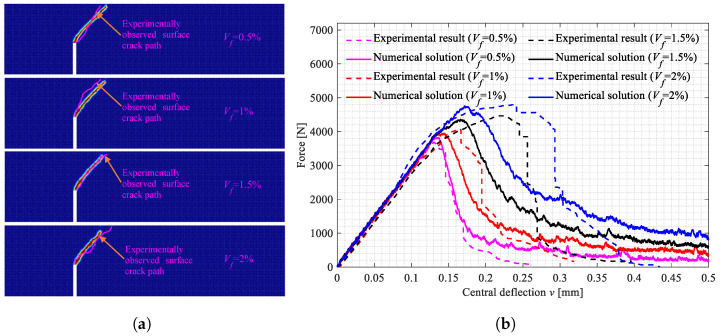
The crack patterns in the beam specimens with $C_{l}=40\;\mathrm{mm}$ and the central deflection-force diagrams. (**a**) Surface crack patterns; (**b**) Central deflection-force diagrams.

**Figure 15:**
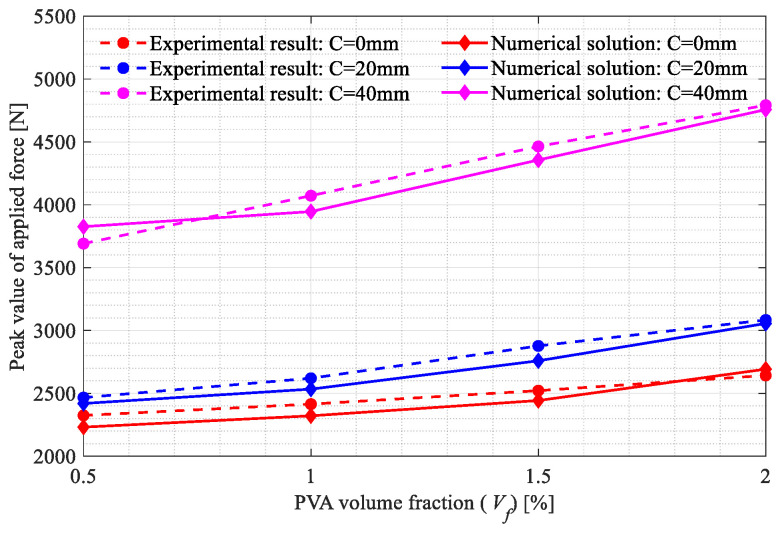
Variations in the peak force values in the simulations versus the volume fractions of PVA fibers in the UHPC-PVAs.

**Figure A1:**
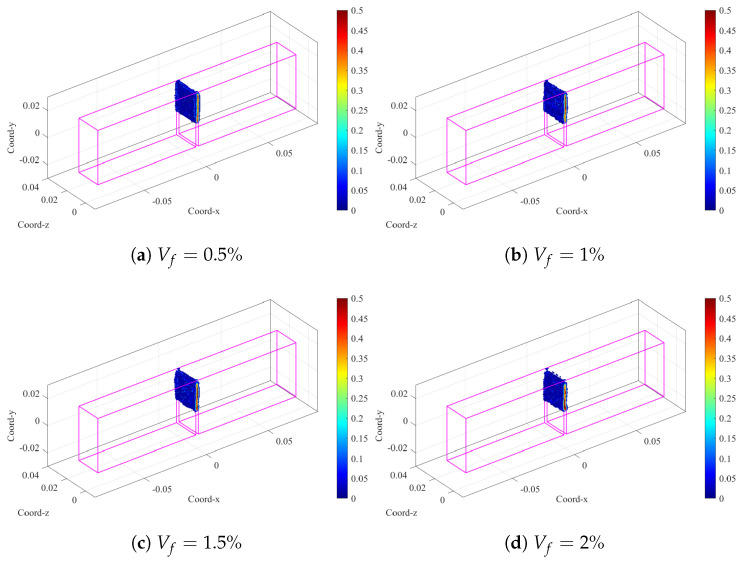
Damage levels in the simulated UHPC-PVA beam specimens with $C_{l}=0\;\mathrm{mm}$.

**Figure A2:**
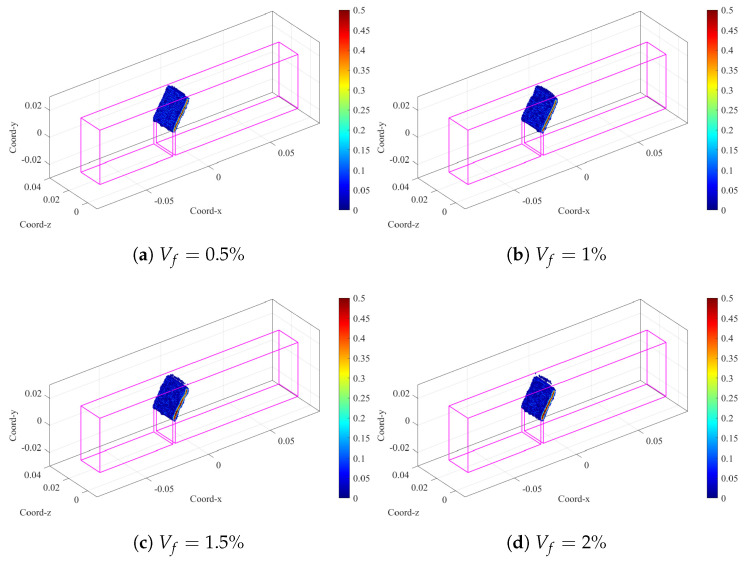
Damage levels in the simulated UHPC-PVA beam specimens with $C_{l}=20\;\mathrm{mm}$.

**Figure A3:**
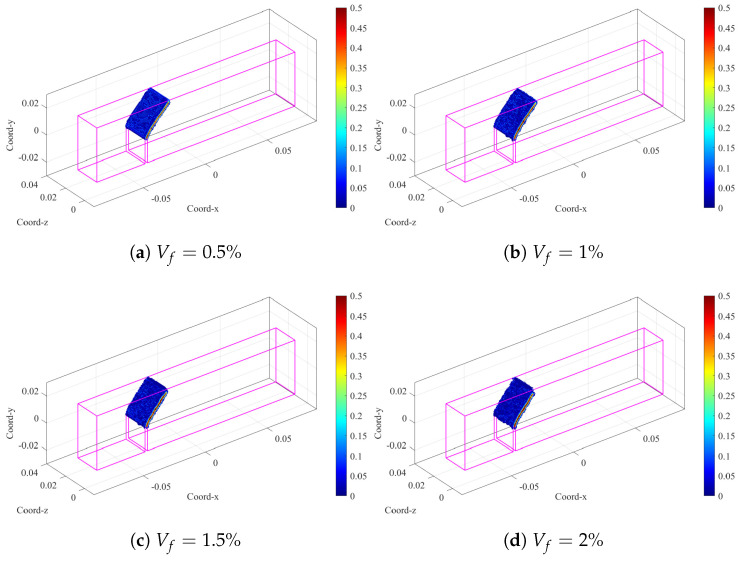
Damage levels in the simulated UHPC-PVA beam specimens with $C_{l}=40\;\mathrm{mm}$.

The surface crack patterns obtained in the simulations are shown in Figure 12a, Figure 13a and Figure 14a, the experimentally observed crack patterns (magenta curves) are also plotted for comparison. The 3D crack surfaces in the simulated specimens are shown in Figure A1, Figure A2 and Figure A3. The corresponding central deflection-force diagrams are plotted in Figure 12b, Figure 13b and Figure 14b. Figure 15 shows the variations of the peak applied force versus the $V_{f}$ values.

The differences between the surface crack patterns obtained in the experiments and numerical simulations may be caused by the random distribution of the PVA fibers in the UHPC-PVA structures. In addition, the damage zones, describing the crack patterns, are thicker in the cases with greater PVA fiber volume fractions, indicating that more bonds are broken in those cases. This is caused by the inconsistency between local deformation and force density due to the reinforcement of the fiber bonds, which is also the reason for the success of the proposed approach in modeling UHPC-PVA materials. Concerning the predicted fracture angles and the peak values of applied force, the simulation results agree well with the experimental results, explaining the adaptability of the proposed modeling approach in describing the failure and fracture behaviours of the produced UHPC-PVA materials and structures.

## 5. Conclusions and Discussions

In this paper, a series of three-point bending tests were carried out with the notched UHPC-PVA beam structures. Different cases were considered to study the effects of the PVA fiber volume fractions on the fracture behaviours of the UHPC-PVAs. Subsequently, in order to track the whole process of fracture advancement in the specimens, a 3D hybrid FE/PD modeling approach was proposed, where the XBB-PD model in conjunction with an energy-based failure criterion [48] was adopted to describe the deformation and failure behaviours of the UHPC-PVAs, removing the limitation on the Poisson ratio in the classical BB-PD model [9]. The comparison between the numerical solutions and the experimental results validates the proposed approach and further demonstrates the reliability of the experimental results.

Based on the above-presented results, we can conclude with the following points:

- With the increasing volume fractions, the PVA fibers show a significantly and linearly increased enhancement to the UHPC-PVAs (see Figure 10); as the PVA fiber volume fraction increased from 0.5% to 2%, the strength of the UHPC-PVA materials increased by 20.7%, 26.3% and 24.3%, respectively, in the cases with $C_{l}=0$, 20 and 40mm;
- In the experimental deflection-force diagrams of the specimens with a greater PVA fiber volume fraction, there exist non-negligible yield behaviors and residual strengths before and after the peak points, reflecting the brittle–ductile transition due to the PVA fiber reinforcement (see Figure 7, Figure 8 and Figure 9);
- Due to the randomness of the fibers and initial defect distribution in the produced UHPC-PVA beam specimens, the crack patterns obtained by simulations show some differences to those from the experiments, which is reasonable;
- The obvious differences between the numerical crack patterns in the cases with the same initial cut position and similar structure strengths (shown in Figure 15) indicate that the proposed approach can reasonably describe the interaction between the fibers and matrix, as well as the reinforcement of the PVA fibers on the UHPC materials.

**Remark:** Although the peak values of applied force predicted by the proposed approach are very close to those obtained by the experiments, the behaviors described by the numerical deflection-force diagrams are different from all the experimental observations excepting the cases with $C_{l}=40\;\mathrm{mm}$ and $V_{f}=0.5\%$. One of the reasons should be the use of the ADR algorithm. As reported in [9,45], the quasi-static solutions obtained by using the ADR algorithm will become closer to the static solutions but this involves greater computing costs. Another reason for the difference should be the linear micro-constitutive relationship used to describe the bond behavior. In fact, such a constitutive relationship is for the prototype microelastic brittle materials. As discussed in [38,39], to accurately characterize the post-peak mechanical behaviors of ductile materials, the linear micro-constitutive relationship is not enough; bilinearity, trilinear or other more advanced non-liear constitutive relationships with more controlling parameters are needed.

Consequently, more efforts in the development of more appropriate constitutive relations and solution algorithms considering both the accuracy and computational efficiency should be made in the future to accurately simulate the mechanical and failure behaviors of the UHPC-PVA materials.

## Figures and Tables

**Table 1:** Mixing ratios of main ingredients used in the production of the UHPC-PVA materials.

Items	Mixing Ratio
Cement	1
Sand	1
Quartz sand	0.3
Silica fume	0.25
Polycarboxylate Superplasticizer	2.5%
Shrinkage reducing agent	2%
Water	0.2
Ice cube	0.02

**Table 2:** Discretization information of the hybrid model for the notched beam specimens.

Case	Number of PD Nodes	Number of FE Elements	Number of Coupling Elements
1 ($C_{l}=0\;\mathrm{mm}$)	22,263	994,880	4320
2 ($C_{l}=20\;\mathrm{mm}$)	80,811	937,760	5440
3 ($C_{l}=40\;\mathrm{mm}$)	135,177	884,720	6480

## Data Availability

The data presented in this study are available on request from the corresponding author.

## Abbreviations

List of Symbols	
$C_{l}$	distance of the initial crack to the middle of the beam specimen
$V_{f}$	PVA fiber volume fraction
ρ	mass density of the material
σ	stress tensor
ε	strain tensor
C	elasticity tensor
$x,\;x^{\prime }$	location vector of material points
u	displacement vector
ξ	relative position vector of two material points
η	relative displacement vector of two material points
$\ddot{u}$	acceleration vector of material point
b	body force density
$H_{x}$	neighborhood associated with the material point x
f	bond force density
c,κ	normal and tangential micro moduli of the bond
ℓ,γ	longitudinal and tangential deformations of the bond
n	unit directional vector along the deformed bond
δ	horizon radius
*w*	strain energy density stored in the deformed bond
$w_{c}$	critical strain energy density of the bond
$G_{c}$	critical energy release rate for mode I fracture
ϱ	characteristic function describing the connection status of bonds
$\varphi_{x}$	damage value at point x
$M^{FE},K^{FE}$	mass and stiffness matrices of FE equations
$N_{u}$	shape functions for displacement
L	differential operator
D	elastic matrix
E,v	Young’s modulus and Poisson’s ratio
$M^{PD},K^{PD}$	mass and stiffness matrices of PD equations
$\ddot{U},\dot{U},U,E,F$	acceleration, velocity, displacement, strain and force vectors
G	non-local strain coefficient matrix
$V_{f}^{g}$	global numerical volume fraction of PVA fibers in the discrete model
$V_{f}^{l_{i}}$	local numerical volume fraction of PVA fibers related to node *i*
$P_{m},P_{f}$	parameters of the matrix and fiber materials
$P_{i}$	average parameter at node *i*
$M^{Coup},K^{Coup}$	mass and stiffness matrices of coupled model
$M,C_{d},K$	fictitious mass, damping and stiffness matrices of the system
Δt	time increment
$M_{ii}$	$i^{th}$principal value of the fictitious mass matrix
$K_{ij}$	elements of the global stiffness matrix
$c_{d}$	system damping coefficient
$K_{t}^{n}$	local diagonal tangent stiffness matrix at the $n^{th}$ iteration
$E_{m},v_{m}$	Young’s modulus and Poisson’s ratio of the matrix materials
$E_{f},v_{f}$	Young’s modulus and Poisson’s ratio of the fiber materials
$h_{m},h_{f}$	geometrically characteristic lengths of the matrix and fiber materials
Δx	grid size
*m*	ratio of the PD horizon radius to the grid size
